# Comparison of different medical treatment options for CRSwNP: doxycycline, methylprednisolone, mepolizumab and omalizumab

**DOI:** 10.1186/2045-7022-5-S4-P41

**Published:** 2015-06-26

**Authors:** Els De Schryver, Lien Calus, Thibaut Van Zele, Claus Bachert, Philippe Gevaert

**Affiliations:** 1 Ghent University Hospital, Department Otorhinolaryngology, Upper Airways Research Laboratory, Ghent, Belgium

## Background

Chronic rhinosinusitis with nasal polyps is hard to treat. The therapeutic effect of doxycycline, oral glucocorticoids, mepolizumab and omalizumab with significant reduction of nasal polyp score was previously investigated by 3 randomized controlled trials. The aim of this study is to compare the effect of these treatments on polyp score, symptom scores and inflammatory parameters.

## Methods

In total, 100 patients were randomly assigned to receive doxycycline for 20 days (n=14), methylprednisolone in decreasing doses for 20 days (n=14), mepolizumab 2 single intravenous injections (n=20), omalizumab 2 to 4 subcutaneous doses (n=15) or placebo (n=37) in three separate clinical double-blind, placebo-controlled trials. Participants were followed for 8 weeks. Endoscopic evaluation of nasal polyp score, assessment of symptom score and measurement of markers of inflammation in nasal secretions and serum occurred at baseline, week 4 and week 8. All treatments were completed at week 4, except for 2 patients who received a fourth subcutaneous dose of omalizumab at week 6.

## Results

All treatment options significantly reduced nasal polyp score as compared to baseline, but not placebo. Methylprednisolone has initially the most dramatic effect on symptom scores, but after cessation of treatment symptom scores worsen progressively and return to baseline after 4 weeks. Mepolizumab, doxycycline and methylprednisolone each had a specific effect on local and systemic inflammatory markers. Omalizumab did not alter eosinophilia or markers of inflammation.

## Conclusions

Omalizumab, mepolizumab and oral doxycycline cause a long-term reduction in nasal polyp size, whereas methylprednisolone initially causes the strongest reduction in polyp size but recurrence occurs earlier. Total symptom scores parallel this trend. Mepolizumab and doxycylin work on eosinophilic and neutrophilic inflammtion in nasal polyps, whereas the effect of methylprednisolone is apparent on eosinophilic markers. Omalizumab did not reduce markers of eosinophilic inflammation, nor neutrophilic inflammation in nasal secretions. The beneficial effect of omalizumab seems to be mediated through a direct effect on IgE or their receptor, rather than through an effect on markers of the eosinophilic cascade.

